# Simple Messages Help Set the Record Straight about Scientific Agreement on Human-Caused Climate Change: The Results of Two Experiments

**DOI:** 10.1371/journal.pone.0120985

**Published:** 2015-03-26

**Authors:** Teresa A. Myers, Edward Maibach, Ellen Peters, Anthony Leiserowitz

**Affiliations:** 1 Center for Climate Change Communication, Department of Communication, George Mason University, Fairfax, Virginia, United States of America; 2 Department of Psychology, The Ohio State University, Columbus, Ohio, United States of America; 3 Yale Project on Climate Change Communication, School of Forestry & Environmental Studies, Yale University, New Haven, Connecticut, United States of America; Mälardalen University, SWEDEN

## Abstract

Human-caused climate change is happening; nearly all climate scientists are convinced of this basic fact according to surveys of experts and reviews of the peer-reviewed literature. Yet, among the American public, there is widespread misunderstanding of this scientific consensus. In this paper, we report results from two experiments, conducted with national samples of American adults, that tested messages designed to convey the high level of agreement in the climate science community about human-caused climate change. The first experiment tested hypotheses about providing numeric versus non-numeric assertions concerning the level of scientific agreement. We found that numeric statements resulted in higher estimates of the scientific agreement. The second experiment tested the effect of eliciting respondents’ estimates of scientific agreement prior to presenting them with a statement about the level of scientific agreement. Participants who estimated the level of agreement prior to being shown the corrective statement gave higher estimates of the scientific consensus than respondents who were not asked to estimate in advance, indicating that incorporating an “estimation and reveal” technique into public communication about scientific consensus may be effective. The interaction of messages with political ideology was also tested, and demonstrated that messages were approximately equally effective among liberals and conservatives. Implications for theory and practice are discussed.

## Introduction

The U.S. National Academies [1], the Intergovernmental Panel on Climate Change [2], the U.S. National Climate Assessment [3], and myriad other leading scientific societies around the world have concluded, with great certainty, that human-caused climate change is occurring. Moreover, a growing body of literature demonstrates that the vast majority of individual climate scientists are also convinced that human-caused climate change is happening. Several methods have been used to estimate the extent of this agreement: both surveys of climate scientists [4–6] and empirical reviews of the peer-reviewed literature [4, 7] estimate the consensus at approximately 97%, with some empirical literature reviews suggesting even higher levels of consensus [8–9].

Yet, relatively few Americans know there is widespread agreement among climate scientists that human-caused climate change is occurring. A 2013 survey showed that only 42% of American adults believe “most scientists think global warming is happening.” Moreover, only about 1 in 5 survey respondents (22%) estimated the level of agreement among climate scientists at more than 80%; the most common response was “don’t know” (28% of the sample) with smaller proportions estimating 61–80% (19%), 41–60% (20%), and even lower estimates (10%) [10]. Several explanations have been offered for why the public doesn’t know about the scientific consensus about human-caused climate change, including “false balance” in news coverage [11] and organized efforts to create an illusion of scientific disagreement [12–15]

Public belief about the level of expert agreement on scientific issues appears to be an important factor in acceptance of scientific propositions across a variety of scientific issues—including humans causing climate change, smoking causing lung cancer, and HIV causing AIDS [16]. In the context of climate change, the evidence suggests that understanding the expert consensus is a “gateway” belief, such that recognition of a high level of scientific agreement about human-caused climate change predisposes people to be more certain that climate change is happening, human-caused, serious, and solvable; in turn, these beliefs are associated with greater support for societal responses to address climate change, and behavior to encourage societal responses [17–19], (but see Kahan [20] for an alternative view). It stands to reason that members of the general public will be less convinced of—and concerned about—climate change if they are under the impression that there is considerable disagreement among climate experts about the reality of human-caused climate change.

In this paper we report the results of two experiments that seek to answer the question: How can scientists and scientific organizations effectively communicate the level of scientific agreement about human-caused climate change? In the first experiment, we tested statements that express the level of agreement through verbal and numeric descriptions, varying the level of precision in the statements to assess how precision influences perceptions of scientific agreement and message credibility. In the second experiment, we tested how asking participants to estimate the level of scientific agreement prior to reading a statement about the scientific consensus influences their subsequent response to the statement.

*Communicating scientific agreement about human-caused climate change*

Recent experimental research has demonstrated that communicating the level of scientific consensus is an effective means of increasing acceptance of science, such that those who viewed messages about the level of scientific consensus on climate change, smoking and lung cancer, and HIV and AIDS were subsequently more likely to report higher perceived consensus and higher acceptance of scientific propositions than those who did not receive such messages [16, 19, 21]. To replicate this finding that messages about the level of scientific consensus can influence perceptions of the level of scientific agreement, we tested a range of statements (both numeric and non-numeric), expecting statements that assert a high level of scientific agreement to increase perceptions of scientific agreement and confidence in those perceptions. Specifically:

*H1*: *Compared to control statements that do not mention the extent of scientific agreement*, *statements that assert a high level of scientific agreement (based on either numeric or verbal descriptors) about human-caused climate change will result in (a) increased estimates of the scientific agreement and (b) increased confidence in those estimates*.

Various approaches to conveying the scientific consensus in numeric and non-numeric terms can be seen in statements made by scientific organizations, political leaders, and others; these different approaches to asserting the scientific consensus are likely to have different impacts. Examples of numeric statements are found on NASA’s climate change web education portal (climate.nasa.gov), “Consensus: 97% of climate scientists agree,” and on a recent Facebook post by President Obama (June 23, 2013).

Examples of non-numeric statements can be found on EPA’s climate change website, “broad agreement exists that climate change is happening and is primarily caused by excess greenhouse gases from human activities” [22] and in a recent National Academies report: “The overwhelming majority of climate scientists agree that human activities, especially the burning of fossil fuels (coal, oil, and gas), are responsible for most of the climate change currently being observed” [1].

How such information is presented may have a large impact on how well it is understood and used [23]. In particular, and drawing upon research that investigates how best to present risk information in medical settings, we examined whether numeric or non-numeric statements would be most effective at increasing perceptions of scientific agreement about human-caused climate change.

Many investigators have examined whether it is preferable to present risk information in non-numeric formats (using qualitative descriptors) or numeric (quantitative) formats. Those who argue for verbal or non-numeric formats contend that presenting information in non-numeric terms is more natural and easily understood (especially by some segments of the public; [24–25]) and guards against communicating unwarranted precision [26–28]. Proponents of numeric descriptions argue that verbal descriptions are inherently imprecise and interpreted with high levels of variability whereas numeric descriptions are better understood [29–32]. In this vein, Peters, Hart, Tusler, and Fraenkel [33] demonstrated that numeric information increased risk comprehension of adverse drug effects and willingness to take a prescribed drug compared to verbal descriptors; this advantage held even among less numerate individuals (see also [34–36]). Additionally, Gurmankin, Baron and Armstrong [37] found that participants were more trusting of and comfortable with numeric descriptions as opposed to verbal ones (see also [38]). In the context of climate change, Budescu, Broomell, and Por demonstrated that the IPCC’s verbal descriptions of probability are widely misunderstood, even when participants were given the technical definition of the probability that each of the verbal descriptions represents [39]. Given these findings, we expected that numeric descriptions of scientific agreement would result in increased perceptions of scientific agreement compared to non-numeric descriptors. We also test whether numeric descriptions influence confidence in those estimations. Specifically:

*H2*: *Numeric scientific agreement messages will result in higher estimates of scientific agreement than non-numeric messages*.

*RQ2*: *Will numeric scientific agreement messages result in greater estimation confidence than non-numeric messages*?

The precision of presented numeric information may also matter. Across a range of topics (prices, distance, percentages, context-free numbers), Janiszewski and Uy reported that more precise numbers (e.g., 4,998 vs. a less precise 5,000) were associated with greater use of those numbers [40]. Similarly, Olson and Budescu demonstrated that participants prefer precision when receiving uncertainty information [41]. Thus, we expected that more precise statements about the level of scientific agreement would lead to higher subsequent estimations of scientific agreement. We also tested whether precision affects estimation confidence, and in what direction.

*H3*: *Compared to less precise statements*, *more precise statements will result in higher estimates of scientific agreement*.

*RQ3*: *Does statement precision also increase estimation confidence*?

Finally, given the politically polarized nature of the climate change opinion landscape [12, 42–43], it may be that political conservatives and liberals respond differently to statements about scientific agreement. Individuals tend to perceive and process information to support pre-existing viewpoints or worldviews, a tendency known as motivated reasoning or cultural cognition [44]. As a result, messages about climate change impacts and scientific agreement on climate change may backfire among political conservatives [18, 44–46]. However, other research has shown the opposite pattern—that messages about scientific agreement on climate change can close the gap between liberals and conservatives [16, 47]. Given these conflicting findings, we tested the interaction of message effect and political ideology on perceptions of scientific agreement and confidence.

*RQ4*: *Does political ideology moderate the effect of statements about scientific agreement*?

## Method

### Ethics Statement

This research was reviewed and approved by the Institutional Research Board at the Office of Research Integrity and Assurance at the lead author’s institution (IRB package #485390). Participants provided consent by first reading a consent form and then choosing one of two options, “I have read this form and agree to participate in this study” or “I do not wish to participate in this study.” Only those who indicated consent by selecting the first option continued the survey and were included in analysis. This consent procedure was approved by the Institutional Research Board.

### Procedure

To test these hypotheses and research questions, we ran a six-condition (five scientific agreement statements plus a control group) post-test only experiment with randomized assignment to condition. The five scientific agreement statements were crossed, such that half included the preface “based on the evidence” prior to the statement and half did not. This “evidence” manipulation did not influence perceptions of scientific agreement or credibility; nor did it interact with the five scientific agreement statements; therefore, the “evidence” conditions were collapsed within each scientific agreement statement (this manipulation was originally included to test whether priming with the evidenced-based nature of climate science would enhance the effect of the scientific agreement messages).

**Fig 1:**
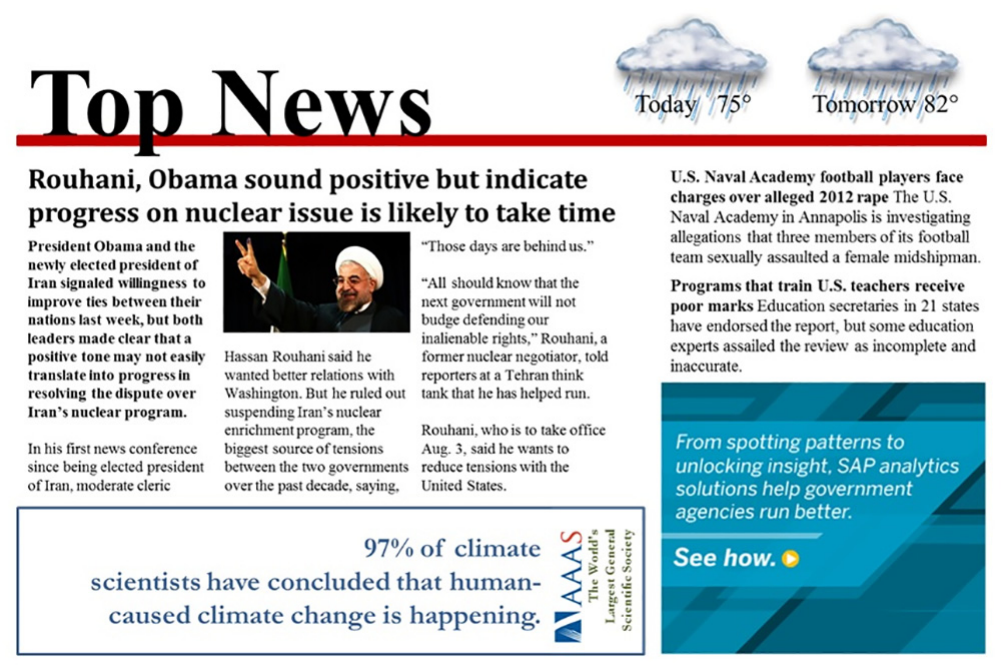
Example of study one stimulus.

An ad—featuring the scientific consensus statement and the American Association for the Advancement of Science [AAAS] logo and tagline—was embedded on a fake news page (see Fig. 1); a control condition featured an ad for a mobile phone in place of the scientific consensus statement. Blurbs from articles in the news at the time of the experiment were used to fill the fake news page. To stimulate engagement with the page, participants were asked to click on areas of the page that made them feel angry and hopeful. Participants (n = 1,116; ≈185 per message condition) then answered a series of survey questions (further description is in S1 Supporting Information, including variables not used in this paper). The ad was not available to view when answering survey questions. The experiment was conducted online with a quota sample (versus a probability sample) of adults who were selected based on known demographic characteristics to resemble the American adult population; participants were recruited through Survey Sampling International [SSI], a vendor that maintains a nationwide panel of people willing to participate in online surveys.

The five statements describing the scientific agreement were: (1) “97.5% of climate scientists…”; (2) “97% of climate scientists…”; (3) “97 out of 100 climate scientists…”; (4) “More than 9 out of 10 climate scientists…”; and (5) “An overwhelming majority of climate scientists…”; all of which ended with the phrase “…have concluded that human-caused climate change is happening.” These messages were chosen to reflect current messages about the climate science consensus and/or because they represented the best available precise estimate.

### Moderator variable

*Political ideology* was measured with a question that asked: “In general, do you think of yourself as…”, with response options of “Very liberal” (coded 1), “Somewhat liberal “(coded 2), “Moderate, middle of the road” (coded 3), “Somewhat conservative” (coded 4), and “Very conservative” (coded 5). Mean political ideology was 2.90, closest to the response option “moderate, middle of the road”, with a standard deviation of 1.14.

### Dependent variables

*People’s estimation of scientific agreement* was measured with a question that asked: To the best of your knowledge, what percent of climate scientists have concluded that human-caused climate change is occurring? Participants could select any number from 0 to 100 on a horizontal slider (*M* = 71.53; *SD* = 24.84).

*Confidence in one’s estimate of the scientific agreement* was measured with a question that asked: “How certain are you about your answer above?” This question was asked immediately beneath the estimation of scientific agreement question. Participants could select any number from 0 to 100 (meaning very certain) on a horizontal slider (*M* = 59.89, *SD* = 31.00).

### Missing data

As is typical in survey data, some people did not respond to all questions asked. To reduce the amount of missing data, we used a hotdeck imputation procedure [48]. To impute values for the missing data, the rows (i.e., respondents) of the survey data file were randomly sorted within sex and education. Any respondent missing on a given variable was assigned the value of a respondent of the same sex and education level nearest to him or her in this randomly sorted data file. Results of the data without imputation were substantially similar and are not included.

### Analysis

All hypotheses and research questions were tested using Ordinary Least Squares regression. Message conditions were coded according to the relevant hypothesis or research question. To test each statement about scientific agreement against the control condition (H1), all scientific agreement statement conditions were coded “1” and the control (a mobile phone advertisement) was coded “0”. To test numeric vs. non-numeric statements (H2), statements #1–4 above (i.e., 97.5%, 97%, 97 out of 100, and more than 9 out of 10) were coded “1” and the non-numeric statement (i.e., #5, an overwhelming majority) was coded “0”. To test message precision (H3), statement #5 (an overwhelming majority) was coded “0” as the least precise, statement #4 (more than 9 out of 10) was coded “1”, statements #2 and 3 (97% and 97 out of 100) were coded “2”, and statement #1 (97.5%) was coded “3.”

## Results

### Descriptive means

**Fig 2:**
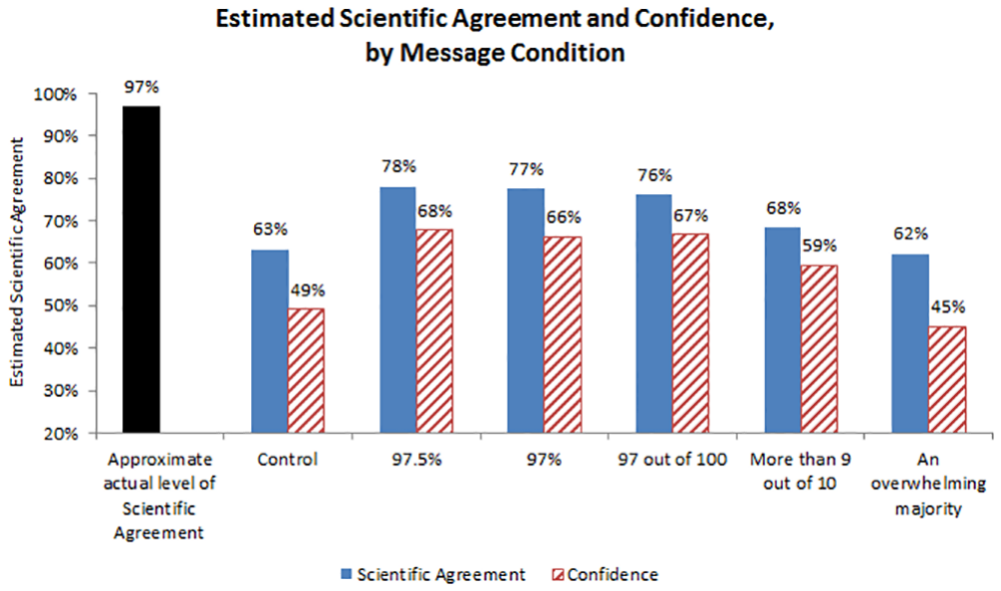
Mean estimated scientific agreement and estimation confidence by message condition, study one.

Means by condition are shown in Fig. 2. Replicating previous studies, those in the control condition substantially underestimated the level of scientific agreement, with participants indicating that 63% of climate scientists have concluded that human-caused climate change is occurring—34% lower than actual levels of scientific agreement. Means of the message conditions ranged from 62% (“an overwhelming majority” condition) to 78% (97.5% condition). Standard deviations across conditions were approximately equal, with the control condition demonstrating the lowest variation between participants (*SD*_*control*_ = 22.35) and those in the “more than 9 out of 10” message condition demonstrating the highest variation (*SD*_*control*_ = 25.21).

Similarly, those in the control condition had relatively low levels of confidence in their estimates, with participants indicating that they were about 49% certain of their estimate. Means in the test conditions ranged from 46% (“an overwhelming majority” condition) to 68% (97.5% condition). Standard deviations across conditions were approximately equal, with responses in the control condition demonstrating the lowest variation between participants (*SD*_*control*_ = 29.13) and those in the 97.5% message condition demonstrating the highest variation (*SD*_*control*_ = 30.88).

### Hypothesis tests

To test H1—that statements which assert a high level of scientific agreement about human-caused climate change will result in increased estimates of the scientific agreement and increased confidence in those estimates—we separately predicted estimated scientific consensus and estimation confidence from the variable that compared our experimental messages vs. control. We found that participants who received a scientific agreement statement had estimates of scientific agreement approximately 9 percentage points higher than those participants in the control condition, *b* = 9.13, *p* <. 001; they also had 12 points higher confidence than those in the control condition, *b* = 12.07, *p* <. 001. Therefore, results were consistent with H1.

To test H2 and RQ2—that numeric scientific agreement statements will result in higher estimates of scientific agreement than non-numeric statements, and whether there is a difference in estimation confidence between numeric and non-numeric statements—we predicted these same two dependent variables from numeric vs. non-numeric messages. Participants in the numeric statement conditions gave estimates of scientific agreement that were approximately 13 points higher than those of participants in the non-numeric message condition, *b* = 12.61, *p* <. 001; they were also approximately 20 points more confident in their estimations, *b* = 19.46, *p* <. 001. Therefore, results were consistent with H2.

To test H3—that more precise statements will lead to higher estimates of scientific agreement—we predicted estimated scientific agreement from statement precision. Results were consistent with H3: more precise statements resulted in higher estimates of scientific agreement, *b* = 5.83, *p* <. 001.

To test RQ3—does statement precision influence estimation confidence?—we predicted estimation confidence from statement precision. Results showed that more precise statements resulted in greater estimate confidence than less precise statements, *b* = 7.58, *p* <. 001.

Finally, to test RQ4—does political ideology moderate the effect of the messages on estimates of scientific agreement and estimation confidence?—we again separately predicted the estimates and estimation confidence from the independent variables (any message vs. control; numeric vs. non-numeric, and level of precision), political ideology, and the interactions of each of these independent variables with ideology (a separate regression was run for each independent variable). When predicting estimated scientific agreement, political ideology did not moderate any of these effects: (a) message vs. control, *b* = 2.54, *p* = .257, (b) numeric vs. non-numeric, *b* = -1.10, *p* = .536, or (c) precision, *b* = -.38, *p* = .574. Political ideology did, however, have a marginally significant moderating effect on estimation confidence in two of the three tests: (a) message vs. control, *b* = 4.15, *p* = .140, (b) numeric vs. non-numeric, *b* = -4.15, *p* = .058, and (c) precision, *b* = -1.63, *p* = .053. Specifically, political conservatives gained marginally less confidence from numeric compared to non-numeric statements and more precise compared to less precise statements than did political liberals, although they did gain significant amounts of confidence in their estimates. For example, the difference in estimation confidence between numeric and non-numeric was 24.20, *p* <. 001 for those who were relatively more liberal (one standard deviation below the mean of political ideology, 1.76). Among conservatives (one standard deviation above the mean of political ideology, 4.04), this same difference was 14.74, *p* <. 001. Similarly, the effect of precision on estimation confidence was strongest for liberals (*b* = 9.50, *p* <. 001), and weaker yet still positive and significant for conservatives (*b* = 5.79, *p* <. 001).

## Discussion

In sum, the results of the first study demonstrated that statements about scientific agreement were effective at increasing American adults’ estimates of the scientific agreement about human-caused climate change as well as their confidence in those estimates. In addition, the results revealed that, numeric descriptions are more effective than non-numeric or verbal descriptions. Number precision may also matter, but the operationalization of precision in the present study was conflated with numeric magnitude and therefore might have been conflated with message strength, such that the more precise, numerical messages may have communicated consensus information in a stronger manner. Furthermore, the more precise numeric messages may have been more attention grabbing in the context of the newspaper advertisement used in our stimulus. Further testing is needed to assess whether the effect we observe is driven by message precision or by message strength.

These findings have important implications as previous work has shown that both perceptions of scientific agreement and confidence about climate beliefs are associated with other meaningful climate beliefs. Perception of scientific agreement is associated with beliefs in various scientific propositions [16] and is likely a gateway for other meaningful climate beliefs [17–19]. Similarly, confidence in understanding of climate change—a distinct, but conceptually similar idea to the confidence in perception of scientific agreement measured in this study—has been shown to polarize belief negatively for those who aren’t convinced of climate science, and positively for those who are [49–50]. Therefore, these findings suggest that efforts by scientific organizations to communicate the extent of the scientific consensus about human-caused climate change have potential to realign public understanding in an appropriate and helpful manner.

Some organizations, however, are currently using qualitative statements to convey the consensus. Our results suggest that qualitative statements may be ineffective; the one qualitative statement that we tested—which is similar to versions that are currently in use by prominent science-based organizations—had no impact relative to our control condition on enhancing public understanding of the scientific consensus. It may be that the ambiguity inherent in this type of verbal description of the level of scientific agreement leaves too much room for differing interpretations. Given that we know that many Americans underestimate the level of scientific agreement among climate scientists, we strongly recommend the use of numeric statements.

Furthermore, our results show that the messages had a similar effect regardless of people’s political ideology—with no evidence that political conservatives reacted negatively to them (although conservatives gained less confidence from our experimental messages than did liberals). This finding suggests that precise, numeric messaging about the high level of scientific agreement can be a productive method for increasing understanding of the scientific consensus among both liberals and conservatives. However, some caution is warranted in generalizing this finding beyond the current context as individuals can also be sensitive to and react to the possibility of being manipulated so that perceived persuasion attempts backfire [51]. It may be that the source of the messages (AAAS) is perceived as neutral and unbiased. Similar messages from sources viewed as political or “liberal” could be perceived differently, and could potentially result in backlash effects among conservatives. Further research in this context should examine source effects.

## Study Two

Study One demonstrated that numeric messages about the level of agreement in the scientific community regarding climate change can be an effective way to increase estimates of the scientific agreement—and people’s confidence in their estimates. In Study Two, we sought to test a potentially complementary approach to debiasing people’s understanding of the scientific agreement about human-caused climate change. In particular, we tested whether eliciting an a priori estimate of the level of scientific agreement before being exposed to a statement about the actual level of consensus would heighten the effect of the statement.

Research on health risk communication has found that numbers are not perceived in a vacuum; whether a number seems large or small, substantial or insubstantial can depend on a number of factors, including comparisons to other numbers [52]. Fagerlin, Zikmund-Fisher, and Ubel tested this possible process in the context of breast cancer communication [53]. They found that women consistently overestimated their risk of breast cancer (average estimate = 46%; actual average risk = 13%). Compared to participants who did not estimate their risk prior to receiving information about their lifetime risk of breast cancer, participants who made an estimate perceived the actual risk of 13% as low and reported feeling relieved about their level of risk. Thus, when people have a general tendency to under- or over-estimate some number, having people make an explicit estimate prior to informing them of the actual number may enhance the debiasing effect of the corrective information by creating an “information gap.” In other words, by revealing to people that they know less about the topic than they may have previously assumed, they may seek to fill that gap with credible new information [54].

Based on these findings and since public perceptions of scientific agreement on climate change are much lower than reality (as discussed above), we expected that asking participants to estimate the level of scientific agreement prior to reading information about actual levels of agreement would create an “information gap” and subsequently increase perceptions of scientific agreement compared to simply being presented the information. Therefore, we hypothesized:

*H5*: *Compared to a no-prior-estimation condition*, *eliciting a prior estimate of scientific agreement before exposing people to a statement about the level of scientific agreement will result in higher subsequent estimates of scientific agreement*, *and greater estimation confidence*.

However, it may be that revealing an “information gap” may backfire if the corrective information is not seen as credible, essentially leading to a process of reactance. This reaction may be especially likely among conservatives, as those who are conservative are less likely to believe in climate change [12, 42–43] and several studies have found that climate messages have the potential to back-fire among conservatives [18, 44, 46]. Thus, we tested whether political ideology influences the effect of eliciting a prior estimate on subsequent estimates of scientific agreement and confidence (RQ2). Furthermore, to test for signs of reactance, we examined whether estimating the level of scientific agreement would result in lower perceptions of message credibility overall and particularly among conservatives.

*RQ6a*: *Will the effect of eliciting an estimate of scientific agreement prior to exposing people to a statement about the level of scientific agreement be moderated by political ideology*?

*RQ6b*: *Does estimating the level of scientific agreement result in lower perceptions of message credibility overall and particularly among conservatives*?

## Method

**Fig 3:**
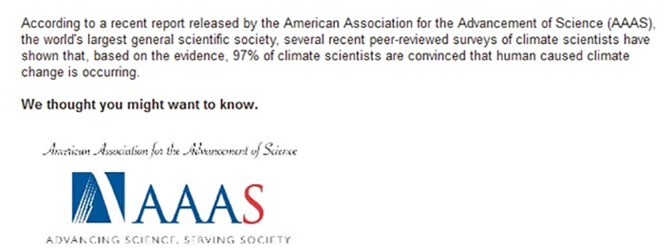
Example of study two stimulus.

To test the proposed hypotheses and research questions, we designed a 2 (prior-estimate, no-prior-estimate) x 4 (3 scientific agreement messages and a control message) condition post-test only randomized controlled experiment. Participants (n = 809, approximately 100 per condition) were again obtained from SSI using the specifications described in Study One. Half of participants (those in the prior-estimate condition) were first asked to indicate what percentage of climate scientists they believe had concluded that human-caused climate change is occurring. They were then asked to read one of four messages from AAAS, which was a short paragraph that contained information about the scientific consensus (see Fig. 3). The other half of participants (the no-prior-estimate condition) were first asked to carefully read one of these four messages from AAAS. Next, all participants were asked a series of survey questions.

Participants in both of these conditions were randomly assigned to one of the four messages from AAAS. The format of the scientific agreement messages is presented in Fig. 3. The first version of the message said “97% of climate scientists are convinced that human caused climate change is occurring” (as shown in Fig. 3). The second version of the message added the words “more than” into the statement, immediately prior to 97% (i.e. “more than 97%”). The third version was identical to the first version, but also offered an explanation for why there is widespread misunderstanding about the level of scientific agreement. Specifically, the explanation offered was: “The news media have contributed to the public’s confusion about the scientific consensus on climate change. The normal act of reporting both sides of the story is misleading in the case of climate science, since nearly all climate scientists are convinced that human-caused climate change is happening. When news media feature the rare dissenting scientist, it gives a false impression that there are two sides to the story. Part of the reason this misperception exists is because of the natural progression of science and the fact that experts’ understanding of the issue has evolved over time as more data was collected. At one time several decades ago there was less agreement among the experts, but as more research was conducted and the evidence accumulated, the vast majority of experts became convinced that human-caused climate change is real.”

The control condition used the same format as the other message conditions, but informed participants that atmospheric CO2 concentrations recently surpassed 400 ppm, without providing any information about the nature of the level of scientific agreement on climate change. In the same manner as the other message conditions, half of participants in the control condition were asked to estimate the level of scientific agreement prior to reading the message, and half were not.

In the analysis, we found no significant differences in scientific agreement based on the three experimental AAAS messages or their interaction with estimate condition. Therefore, we collapsed the message conditions to enhance power and guard against case-category confounds (e.g., believing the effect is due to a *type* of message, when the effect *may be* due to one particular message [55–56]; Jackson, O’Keefe, & Jacobs, 1988; Slater, 1991). Thus, in effect, we simplified the experiment to a 2 (prior-estimate, no-prior-estimate) x 2 design (scientific agreement message, control).

### Measures

Measures were identical to those in Study One, with the addition of message credibility. Additional measures (with their descriptive means) that were not the focus of the present paper can be found in S1 Supporting Information.

*Message credibility* was measured with five items that asked participants their perceptions of the information presented. Participants were asked to rate their answers to: “How would you describe the climate change information that you just read?” on seven-point (0–6) semantic differential scale anchored by the following terms (1) Fair:Unfair; (2) Biased:Unbiased; (3) Tells the whole story:Doesn't tell the whole story; (4) Accurate:Inaccurate; and (5) Can't be trusted:Can be trusted. Responses were averaged to create the measure of message credibility, with the mean at approximately the mid-point of the scale (*M* = 3.69, *SD* = 1.45, α = .79).

### Analysis

As in Study One, all hypotheses and research questions were tested using Ordinary Least Squares regression. Messages were coded into two variables: estimation condition and scientific agreement message condition. *Estimation condition* was coded “0” for those who made no prior estimate and “1” for those who made a prior estimate. *Scientific agreement message condition* was coded “0” for those who read the control message (about CO_2_ reaching 400 ppm) and “1” for those who read a scientific agreement message. The interaction between estimation condition and scientific agreement message condition was also included in the analyses.

## Results

### Descriptive means and associations

**Fig 4:**
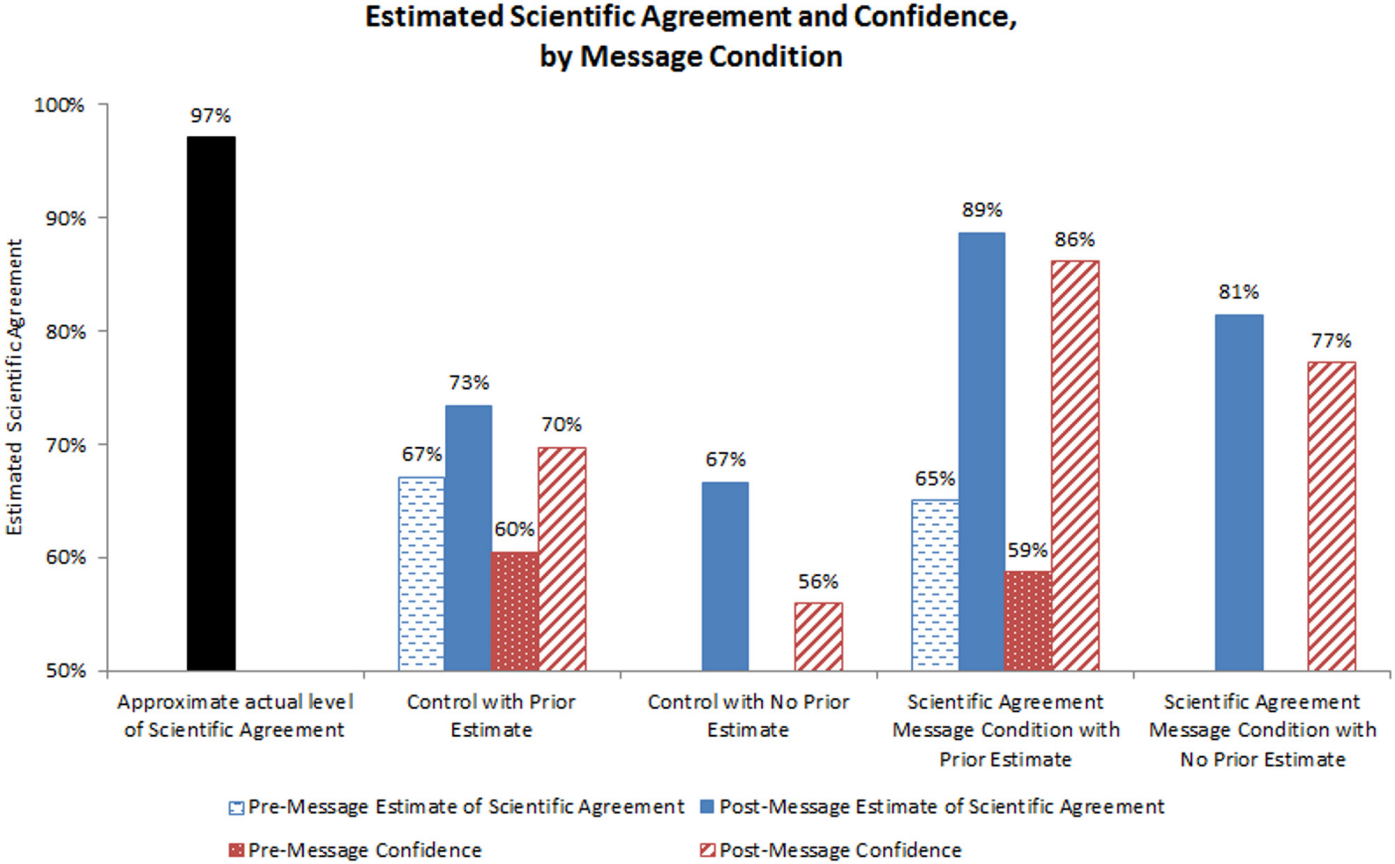
Mean estimated scientific agreement and estimation confidence by message condition, study two.

Means by condition are shown in Fig. 4. As in Study 1, control respondents who had not been provided the actual level of scientific agreement substantially underestimated the level of scientific agreement. In particular, those in the prior-estimate condition made average prior estimates of scientific agreement of 67%. Similarly, no-prior-estimate control respondents estimated an average of 67% agreement. Thus, when respondents were given no information about the level of scientific agreement their perception of the level was approximately 30 points lower than actual levels of scientific agreement.

Among participants in the prior-estimate scientific agreement message condition, 74% increased their estimate of perceived scientific agreement after reading the scientific agreement message, 16% of participants stayed at the same level, and 10% of participants decreased their estimate of scientific agreement. Increases in perceived scientific agreement ranged from 1% to 97%, decreases ranged from -1% to -64%. The average change in perceived scientific agreement among those in the prior-estimate condition from pre- to post-message reading was +23 points (as the item was measured from 0–100, points can also be interpreted as percentages).

Among those participants in the prior-estimate condition, participants’ average confidence in their prior estimates of scientific agreement was 60. There was a strong positive relationship between the magnitude of participants’ prior estimates of scientific agreement and their confidence in their estimates (*b* = .56, *p* <. 001). In other words, prior to receiving any information, those who estimated higher levels of scientific agreement were more confident in their estimates than those who estimated lower levels of scientific agreement.

Among all participants, confidence in their estimates of scientific agreement after reading the message (including those in the control conditions) averaged 77 and was again strongly associated with their post-message scientific agreement estimates (*b* = .65, *p* <. 001). Among those in the prior-estimate condition who read a scientific agreement message, pre-post changes in estimated scientific agreement were positively associated with changes in estimation certainty before vs. after reading the scientific agreement message, *b* = .48, *p <*. 001. Among those who increased their estimates of scientific agreement, the average increase in estimation certainty was 31 points, whereas among those who decreased their estimates, there was no average change in estimation certainty (mean increase = .23, one-tailed t-test, *t* = .04, *p* = .97).

### Hypothesis tests

To test H5—eliciting an estimate of scientific agreement prior to exposing participants to a statement about the scientific consensus will result in higher levels of post-message estimated scientific agreement and estimation confidence—we predicted post-message estimated scientific agreement and confidence from estimation condition. Results showed that participants in the prior-estimate condition provided post-message estimates of scientific agreement that were 7 points higher (*b* = 7.18, *p* <. 001) and confidence in their ratings that were approximately 9 points more (*b* = 8.93, *p* <. 001) than those in the no-prior-estimate condition. Therefore, results were consistent with H5.

To test RQ6a—will the effect of eliciting an estimate of scientific agreement prior to exposing participants to a message about scientific agreement on post-message estimated scientific agreement and confidence be moderated by political ideology?—we predicted post-message estimated scientific agreement and confidence from estimation condition, scientific agreement message condition, political ideology and the interaction of estimation condition by political ideology. There was no evidence that political ideology moderated the effect of making a prior estimate on post-message perceptions of scientific agreement (*b*_interaction_ = .86, *p* = .571) or on estimation confidence (*b*_interaction_ = 1.23, *p* = .496).

To test RQ6b—does estimating the level of scientific agreement result in lower perceptions of message credibility overall, and especially among conservatives—we predicted message credibility from estimation condition. There was no evidence that providing an estimate resulted in lower levels of perceived message credibility, in comparison to not providing an estimate (*b* = .11, *p* = .343). Next, we added political ideology and the interaction between estimation condition and political ideology to the model. Results showed no support for the idea that the effect of eliciting an estimate on perceptions of message credibility differed by participant’s political ideology, (*b*_interaction_ = .01, *p* = .956).

## Discussion

Replicating Study One, the results of Study Two provided further evidence that simple clear messages about the level of scientific agreement increase subsequent estimates of the scientific consensus as well as confidence in those estimates. Additionally, Study Two demonstrated that asking people to state their current estimate of the scientific consensus before sharing with them the correct information accentuates the effect of providing correct information on subsequent estimates of the consensus, as well as their confidence in those estimates. Specifically, the average accentuation effect was approximately seven percentage points for estimates of the scientific consensus, and ten points for estimation confidence. We found no evidence of reactance in response to eliciting a prior estimation; specifically, doing so did not decrease perceptions of message credibility among conservatives. These findings are in line with previous research that has shown that the comparative context within which numbers are presented is a critical factor.

**Fig 5:**
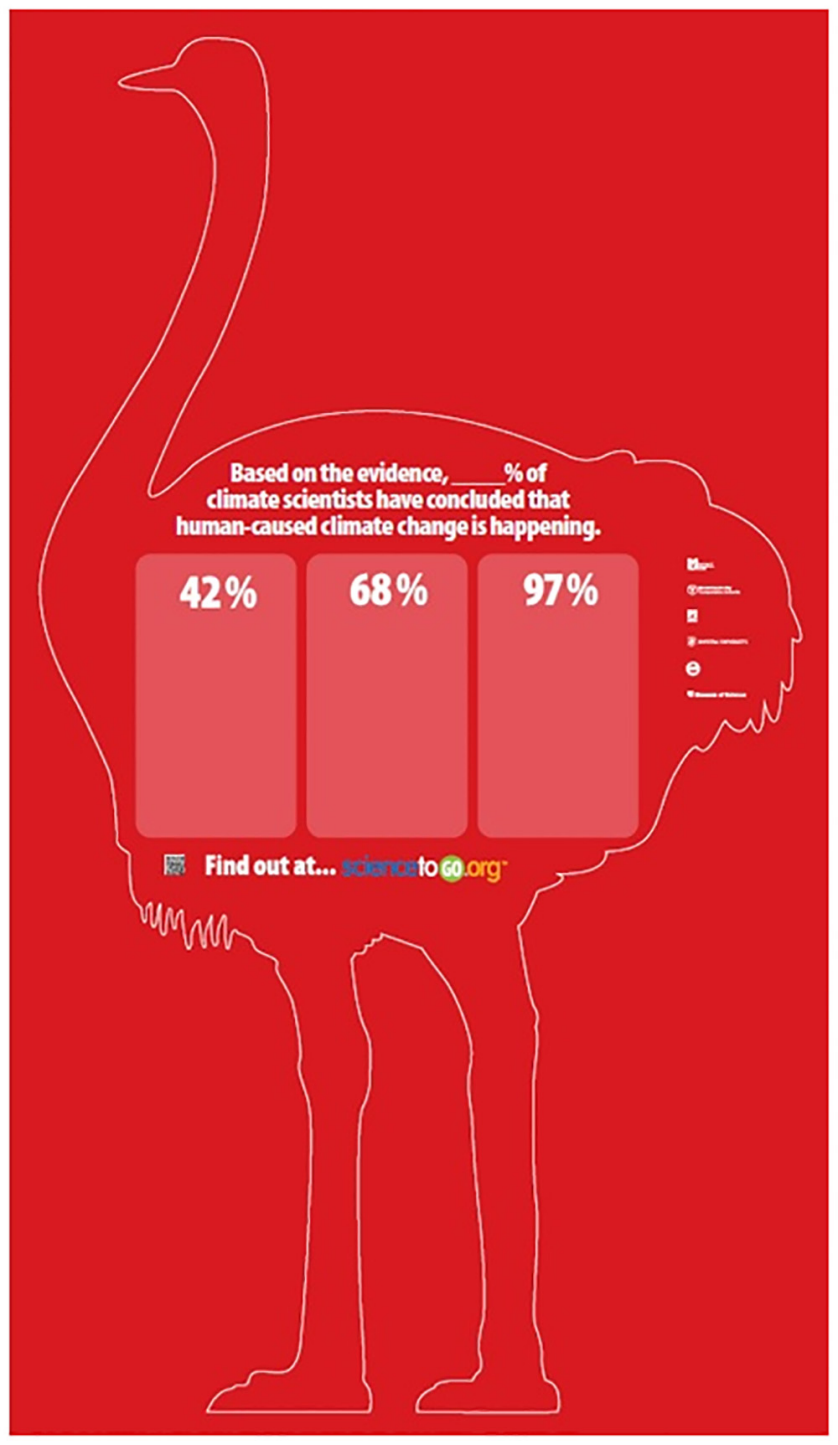
Example of a public design incorporating the “estimation and reveal” finding from study two.

Scientific organizations—and individual scientists—who wish to set the record straight about the scientific consensus concerning human-caused climate change may wish to consider how to include this element of “estimation and reveal” in their designs. For example, prior to revealing the true level of scientific agreement (with the evidence of how that level was assessed), individual scientists could ask their audience to write down what they believe the current level of scientific agreement to be. Fig. 5 shows a creative approach of the estimation and reveal method that is currently being employed in a Boston-based subway campaign.

## Conclusion

Taken together, these two studies provide solid evidence that simple clear messages can be used to increase American’s understanding of the scientific consensus about human-caused climate change and their confidence in that assessment. A single exposure to these messages had a substantial impact on debiasing participants’ under-estimates of the scientific consensus.

It is also important to note that even the most effective messages tested—under the most effective condition (when a prior estimate was elicited)—did not fully result in perceptions identical to the reported level of scientific agreement. The most effective messages led to an 89% estimate of scientific consensus (at 86% level of confidence)—approximately 8 points lower than the actual 97% level of scientific consensus. It is unlikely that a single exposure to a scientific agreement message will result in complete belief updating; however, it is likely that multiple repetitions of the message over time, from multiple trusted sources, will enhance the debiasing effectiveness of these messages. Simple clear messages, repeated often, by a variety of trusted sources is an effective framework for public communication [57].

These studies provide no insight into the degree to which these communication effects are robust. For example, how long do the effects endure? How resistant are they to counter claims? Under what conditions do these scientific agreement messages influence other related climate beliefs, like certainty that human-caused climate change is occurring and support for action? Furthermore, in these experiments the scientific consensus message communicator was the AAAS—a large and trusted scientific society; would a less trusted messenger result in different (or boomerang) effects? Given the potential importance of setting the record straight on the scientific consensus about human-caused climate change, these are questions that warrant answers through further research, especially in experimental and longitudinal contexts.

We conclude by urging scientists and scientific organizations to continue, renew, or start efforts to convey the scientific agreement on climate change, and we offer two practical recommendations based on our findings. Statements about the scientific consensus should:

1. Use numeric, rather than verbal, descriptions of scientific agreement.
2. If possible, incorporate the “estimation and reveal” method.

## Supporting Information

S1 Dataset The dataset for study one. (SAV)

S2 Dataset The dataset for study two. (SAV)

S1 Supporting Information Supplemental material for these studies. (DOCX)
