# Correction: Simple Messages Help Set the Record Straight about Scientific Agreement on Human-Caused Climate Change: The Results of Two Experiments

**DOI:** 10.1371/journal.pone.0133103

**Published:** 2015-07-15

**Authors:** The PLOS ONE Staff

## Notice of Republication

This article was republished on June 23, 2015, to replace copyrighted or confidential material. Please download this article again to view the correct version.
