# Supplementary material for: Simple Messages Help Set the Record Straight about Scientific Agreement on Human-Caused Climate Change: The Results of Two Experiments
Source: PLoS One. 2015 Mar 26;10(3):e0120985. doi: 10.1371/journal.pone.0120985 (PMC4374663; doi:10.1371/journal.pone.0120985)
Supplement: S1 Supporting Information — (DOCX) [file pone.0120985.s003.docx]

**Supplemental Material**

This supplemental material displays means of other variables not central to our original analysis. Furthermore, the direct and indirect effects of message conditions on these variables are displayed.

*Measures*

*Belief Certainty that Climate Change is Happening* was measured with a branching set of questions that first asked: Climate change refers to the idea that the world’s average temperature has been increasing over the past 150 years, may be increasing more in the future, and that the world’s climate may change as a result. What do you think: Do you think that climate change is happening? Participants could respond “yes,” “no,” or “don’t know.” If participants responded “yes” they were then asked: How sure are you that climate change is happening? Response options were not at all sure,” “somewhat sure,” “very sure,” and “extremely sure.” If participants answered “not to the first question, they were asked: How sure are you that climate change is not happening? Answers were merged to create a 9 point scale ranging from 0 “extremely sure climate change is not happening” to 8 “extremely sure climate change is happening” (*M* = 6.12; *SD* = 1.92).

*Belief that Climate Change is Human-Caused* was measured with a question that asked: Assuming climate change is happening, do you think it is… (a) caused entirely by human activities [coded “5”], (b) caused mostly by human activities, (c) caused about equally by human activities and natural changes in the environment, (d) caused mostly by natural changes in the environment, (e) caused entirely by natural changes in the environment, or (f) none of the above because climate change is not happening [coded “0”]. (*M* = 3.23; *SD* = 1.08).

*Perception of the Harmfulness of Climate Change* was assessed with two questions. The first asked: When do you think climate change will start to harm people in the United States? Response options were: they are being harmed right now, in 10 years, in 25 years, in 50 years, in 100 years, and never (reverse coded). The second asked: How much do you think climate change will harm your family? Response options were: not at all, only a little, a moderate amount, a great deal, and don’t know (excluded from analysis). These two measures were correlated, *r* = .511, *p* < .001. Answers to both questions were standardized and averaged to create a *harmful* score (*M* = 0; *SD* = .87).

*The Level of the Effort that the United States Should Make* *to Address Climate Change* was assessed with one question that asked: How big of an effort should the United States make to reduce climate change? Response options were “no effort; ” “a small effort even if it has small economic costs;” “a moderate effort, even if it has moderate economic costs;” and “a large-scale effort, even if it has large economic costs” (*M* = 2.92; *SD* = .95).

***Means of Belief Certainty, Human Causation, Harmfulness, and US Effort***

| **Table S1. Means of Climate Change Beliefs by Message Condition, Study One** | | | | | | | | |
| --- | --- | --- | --- | --- | --- | --- | --- | --- |
|  |  | Belief Certainty |  | Human Causation |  | Harmfulness |  | US Effort |
| Control |  | 6.08 |  | 3.18 |  | -.07 |  | 2.91 |
| 97.5% |  | 6.14 |  | 3.19 |  | .08 |  | 2.92 |
| 97% |  | 6.13 |  | 3.27 |  | -.03 |  | 2.95 |
| 97 out of 100 |  | 6.16 |  | 3.24 |  | .07 |  | 2.97 |
| More than 9 out of 10 |  | 6.24 |  | 3.29 |  | .05 |  | 2.93 |
| Overwhelming Majority |  | 5.99 |  | 3.18 |  | -.13 |  | 2.84 |

| **Table S2. Means of Climate Change Beliefs by Message Condition, Study Two** | | | | | | | | |
| --- | --- | --- | --- | --- | --- | --- | --- | --- |
|  |  | Belief Certainty |  | Human Causation |  | Harmfulness |  | US Effort |
| Control with No-Prior- Estimate |  | 6.41 |  | 3.44 |  | -.03 |  | 3.00 |
| Control with Prior-Estimate |  | 6.38 |  | 3.36 |  | -.06 |  | 2.89 |
| Scientific Agreement Message with No-Prior-Estimate |  | 6.32 |  | 3.39 |  | .01 |  | 3.00 |
| Scientific Agreement Message with Prior-Estimate |  | 6.56 |  | 3.37 |  | .02 |  | 3.04 |

| ***Direct and Indirect Effects of Messages on Belief Certainty, Human Causation, Harmfulness, and US Effort***  **Table S3. Direct Effects of Conditions on Belief Certainty, Human Causation, Harmfulness, and US Effort** | | | | | | | | | |
| --- | --- | --- | --- | --- | --- | --- | --- | --- | --- |
|  |  |  | Belief Certainty |  | Human Causation |  | Harmfulness |  | US Effort |
| *Study One* | |  |  |  |  |  |  |  |  |
|  | Any Scientific Agreement Statement vs. Control |  | .051 |  | .059 |  | .079 |  | .009 |
|  | Numeric vs. Non-Numeric |  | .173 |  | .076 |  | .165* |  | .107 |
|  | Precision |  | .035 |  | .003 |  | .055* |  | .033 |
| *Study Two* | |  |  |  |  |  |  |  |  |
|  | Estimation vs. No Estimation |  | .014 |  | -.128 |  | -.079 |  | -.082 |
|  | Scientific Agreement Message vs. Control |  | -.297* |  | -.225** |  | .102 |  | -.099 |

**p* < .01, ***p* < .001; *Note: Entries are unstandardized regression coefficients, predicting the dependent variables of belief certainty, human causation, harmfulness, and US Effort.*

| **Table S4. Indirect Effects of Conditions on Belief Certainty, Human Causation, Harmfulness, and US Effort through Scientific Agreement** | | | | | | | | | |
| --- | --- | --- | --- | --- | --- | --- | --- | --- | --- |
|  |  |  | Belief Certainty |  | Human Causation |  | Harmfulness |  | US Effort |
| *Study One* | |  |  |  |  |  |  |  |  |
|  | Any Scientific Agreement Statement vs. Control |  | .176* |  | .100* |  | .018* |  | .067* |
|  | Numeric vs. Non-Numeric |  | .245* |  | .134* |  | .079* |  | .089* |
|  | Precision |  | .117* |  | .064* |  | .037* |  | .042* |
| *Study Two* | |  |  |  |  |  |  |  |  |
|  | Estimation vs. No Estimation |  | .161* |  | .093* |  | .077* |  | .081* |
|  | Scientific Agreement Message vs. Control |  | .343* |  | .198* |  | .163* |  | .173* |

*Note: Entries are indirect effects of the message condition (the rows of the table) on the outcomes of belief certainty, human causation, harmfulness, and US Effort, through the mediation of perceptions of scientific agreement. Thus, the top left entry of .176 indicates that viewing a scientific agreement message significantly increased climate change belief certainty through perceptions of scientific agreement. All indirect effects were estimated with Hayes’ (2013) PROCESS macro. 95% confidence intervals were constructed for each indirect effect, one star indicates that zero was not contained within the confidence interval, and therefore that p < .05.*

*References*

Hayes, A. F. (2013). *Introduction to mediation, moderation, and conditional process analysis: A*

*regression-based approach.* The Guilford Press: New York.
